# Some Remarks on Gymnastic Exercises

**Published:** 1827-12

**Authors:** J. Macartney


### GYMNASTIC EXERCISES.

*Some Remarks on Gymnastic Exercises.* By J. Macartney, M.D.

In your Journal for October, there is a case communicated by Mr. Rose, which, if passed without remark, may be the means of prejudicing some persons' minds against the use of gymnastic exercises; and, as I have had the opportunity of knowing the real effects of such exercises, in consequence of having a gymnasium for the use of my pupils, I feel it necessary to say I am satisfied that, so far from the voluntary and methodical exertions of the muscles being likely to produce strains, dislocations, fractures, or herniæ, they form the greatest security against such accidents. In almost all the injuries to the bones or joints, and the yielding in the openings of the abdomen, external force is accompanied with either feebleness or with a sudden and undirected muscular contraction. The power in the animal machine of resisting external violence depends as much on the regulation of muscular efforts by volition, as the success of the individual in any moral emergency depends on the direction of his mental energy, or what is called presence of mind.

It is of the utmost consequence that all teachers of gymnastics, and all who undertake the training of youth, should carefully distinguish between voluntary muscular efforts and fixed and fatiguing positions, as the effects are the very opposite to each other.

It appears to me that the circumstance to which Mr. Rose ascribes the accident which terminated fatally, was not, properly speaking, a gymnastic exercise, but one of those positions which are erroneously thought to prepare persons for dancing. The patient also evidently possessed a weak and irritable constitution, and her sufferings accumulated from a slight degree of inflammation in the beginning, and such as in most persons would have been attended necessarily with no serious consequence. The case, therefore, cannot properly be adduced as an effect of the practice of gymnastics, and might have ensued from the common exercise of walking, and probably never would have existed if the lady had been early trained to the voluntary practice and exertion of her muscles.

You will oblige me by inserting the preceding observations in your Journal.

*Dublin; October*, 1827.

P. S.—A friend has just communicated to me the following fact:—namely, that at Mr. Fellenberg's school at Hoffwel, during six years that he had resided there, not a single accident had been the consequence of gymnastic exercises, although they were employed by about one hundred young people every day.

